# Meta‐analysis of immune‐related adverse events of immune checkpoint inhibitor therapy in cancer patients

**DOI:** 10.1111/1759-7714.13541

**Published:** 2020-07-08

**Authors:** Peng Song, Dingding Zhang, Xiaoxia Cui, Li Zhang

**Affiliations:** ^1^ Department of Respiratory Medicine, Peking Union Medical College Hospital Chinese Academy of Medical Science & Peking Union Medical College Beijing China; ^2^ Central Research Laboratory,Peking Union Medical College Hospital Chinese Academy of Medical Science & Peking Union Medical College Beijing China

**Keywords:** Cancer, immune checkpoint inhibitor (ICI), immune‐related adverse events (irAEs)

## Abstract

**Background:**

Immune checkpoint inhibitors (ICIs) have significant clinical efficacy in the treatment of non‐small cell lung cancer (NSCLC); however, the incidence of immune‐related adverse events (irAEs) of up to 50% has prevented their widespread use. With the increase in the use of ICIs alone or as combination therapy, clinicians are required to have a better understanding of irAEs and be able to manage them systematically. In this study, we aimed to assess the incidence of irAEs associated with ICIs.

**Methods:**

We searched PubMed, Embase, and the Web of Science databases, and also included relevant literature references to widen our search. The relevant data with inclusion criteria were performed using RevMan 3.6.0 for meta‐analysis. We undertook a systematic literature search which included published data up to December 2019.

**Results:**

Overall, 147 articles and 23 761 cancer patients with 11 different ICI treatment‐related (grade 1–5 and 3–5) irAEs were included in the study. There were 46 articles on pembrolizumab (6598 patients), 27 on nivolumab (3576 patients), 13 on atezolizumab (2787 patients), 12 on avelumab (3213 patients), 10 on durvalumab (1780 patients), 22 on ipilimumab (4067 patients), eight on tremelimumab (1158 patients), three on JS001 (223 patients), four on camrelizumab (SHR‐1210) (178 patients), one on sintilimab (96 patients), and one on cemiplimab (85 patients). Grade 1–5 irAEs were: cytotoxic T lymphocyte antigen 4 (CTLA‐4) (82.87%), programmed cell death 1 (PD‐1) (71.89%), and programmed cell death ligand‐1 (PD‐L1) (58.95%). Subgroup analysis was: Avelumab (44.53%), durvalumab (66.63%), pembrolizumab (67.25%), atezolizumab (68.77%), nivolumab (76.25%), Ipilimumab (82.18%), and tremelimumab (86.78%). Grade 3–5 irAEs were: CTLA‐4 (27.22%), PD‐1(17.29%), and PD‐L1(17.29%). Subgroup analysis was: Avelumab (5.86%), durvalumab (13.43%), atezolizumab (14.45%), nivolumab (15.72%), pembrolizumab (16.58%), tremelimumab (22.04%), and ipilimumab (28.27%).

**Conclusions:**

This meta‐analysis confirmed that anti‐PD‐1 and anti‐PD‐L1 inhibitors had a lower incidence of irAEs compared with anti‐CTLA‐4 inhibitors.

## Graphical abstract

This becomes very challenging when oncologists must make decisions among many therapies with similar efficacy and / or specific toxicity characteristics. The advantage of meta‐analysis in this study is that it can reduce publication bias and identify obvious results with higher efficiency. Due to the combination of smaller and larger studies, the effective sample size will be greatly increased. The results of this meta‐analysis can help oncologists choose the type of immune checkpoint inhibitor when deciding on an ICI plan and when planning to use ICI for future research.

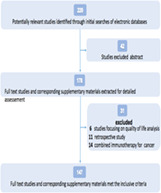

## Introduction

Immune checkpoint inhibitors (ICIs) act on cell surface checkpoint proteins to detect and destroy cancer cells through the autoimmune system, and can effectively be used to treat many types of malignant tumors. However, such treatment could lead to immune‐related adverse events (irAEs).^1^ ICIs include monoclonal antibodies (mAbs) against programmed cell death receptor‐1 (PD‐1), PD‐1 ligand (PD‐L1), and cytotoxic T lymphocyte‐associated antigen‐4 (CTLA‐4), which have been approved for the treatment of advanced malignant tumors. PD‐1 and CTLA‐4 belong to the CD28 superfamily. PD‐1 transmits a negative signal to T cells after binding to one of its two ligands (PD‐L1 or PD‐L2).^2^ When PD‐1 binds to its ligand, it inhibits the kinase involved in T cell activation, which allows tumor cells to escape immune detection and attack.^3^ ICIs have been approved by the US Food and Drug Administration (FDA) for the treatment of advanced malignancies such as melanoma, non‐small cell lung cancer (NSCLC), renal cell carcinoma (RCC), urothelial carcinoma, head and neck squamous cell carcinoma, and Hodgkin’s lymphoma. Because ICIs can activate T cells, its adverse reactions are different from traditional cytotoxic chemotherapy and are immune‐mediated responses such as colitis, hepatitis, thyroiditis, pituitary inflammation, pneumonia, pericarditis, hypothyroidism, nephritis, fatigue, and rash.^4^

ICIs are constantly experiencing exploration from second‐line to first‐line therapies. After 2011, following numerous clinical trials and studies, the FDA successively approved pembrolizumab, nivolumab (PD‐1), atezolizumab, avelumab, durvalumab (PD‐L1), followed by the approval of ipilimumab, and currently tremelimumab (CTLA‐4) is undergoing a large number of clinical trials. From 2017 to 2018, China's immunization research progressed rapidly, and PD‐1 ICIs (JS001, SHR‐1210, and sintilimab) became available in China. Cemiplimab has been approved by the FDA and has become the world's third type of PD‐1 ICI. These immune drugs have shown good responses to malignant tumors, but their accompanying irAEs should not be ignored.

With the deepening of clinical research to clinical practice, tumor immunotherapy has accumulated more and more research data in the treatment of indications, the selection of biomarkers, and the prevention and treatment of AEs related to immunotherapy. Here, we conducted a comprehensive meta‐analysis of single drug immunotherapy for different cancer types and different ICI treatment‐related AEs to provide an effective data support for future clinical immunotherapy decisions.

## Methods

## Literature search and eligibility criteria

A systematic literature search for relevant articles was conducted which included single‐ or two‐arm clinical trials of immunotherapy with a single drug for malignant tumors. Inclusion criteria were as follows: the study subjects were patients with malignant tumors receiving ICI monotherapy, regardless of age, gender, nationality, and ethnicity. Interventions: the experimental group consisted of patients who had received ICI monotherapy and the control group of patients who had received conventional chemotherapy. Outcome indicators were evaluable ICI‐related AEs. Document exclusion criteria were: (i) unable to obtain full‐text, or repeated publication studies; (ii) documents with incomplete data or lack of original extractable data; and (iii) summary or no indicators for evaluation.

**Search strategy**

The database searches included PubMed, Web of Science, and Embase. The single‐ or two‐arm clinical trials were published in English from January 2007 to May 2019. The combination of key words and free words to search included specific ICI drug names: Pembrolizumab, avelumab, nivolumab, atezolizumab, durvalumab, ipilimumab, tremelimumab, JS001, SHR‐1210, sintilimab, and cemiplimab, and keywords were immunological checkpoints (PD‐1, PD‐L1, CTLA‐4) to search for relevant English studies of immunotherapy. A supplementary literature search was also undertaken which included the references in the literature.

### Document screening method

Two researchers independently (XY Cui, P Song) conducted a literature search and screening according to the Quality of Reporting of Meta‐Analyses (QUORUM). The literature screening was based on the predetermined inclusion or exclusion criteria. First, the researchers read the title and abstract, later, read the full text to screen the qualified documents. In case of disagreement, the research team collectively discussed the solution.

### Data extraction

Data were extracted according to the same data extraction table. The final results were crosschecked. The extracted data included mainly treatment‐related and immune‐mediated research data and the following data were extracted: PMID, first author, study year, cancer type, region, the total number of trials, enrollment included in safety analysis, study phase, treatment plan, common AE assessment version, the total number of grade 1–5 ICI‐related AEs and grade 3–5 ICI‐related AEs, specific data and frequency of various AE reports.

### Statistical analysis

#### Combined effect values

The incidence of AEs in the original study was calculated by recording the sample size of the original study and the number of AEs. The inverse variance method was used to combine the incidence of AEs in a single study to draw the forest map. A fixed‐effect or a random effect model was selected based on the heterogeneity test result. When the heterogeneity between studies was larger and the heterogeneity source could not be found by stratified analysis, the combined results of individual study AEs should be carefully interpreted.

#### Heterogeneity test

The interstudy heterogeneity (I^2^) was calculated using the DerSimonian‐Laird estimate method; the heterogeneity between studies was analyzed by chi‐square test. When *P* > 0.10, I^2^ ≤ 50%, there was no heterogeneity between them; when *P* ≤ 0.10, I^2^ > 50%, there was a larger heterogeneity between the studies.

#### Publication bias test

By drawing a funnel chart and observing its symmetry, the risk of publication bias was initially determined. If the funnel chart showed symmetry, it indicated that there was no publication bias; if the funnel chart showed asymmetry, it indicated that there was a publication bias. At the same time, the symmetry of the funnel chart was judged by a rank correlation test.

#### Hierarchical analysis

We performed a stratified analysis according to specific ICI drug varieties. Statistical analysis was conducted with the meta‐analysis package of R software (3.6.0), using the metaprop, forest, funnel, and metabias commands.

## Results

### Literature screening

**Figure 1:**
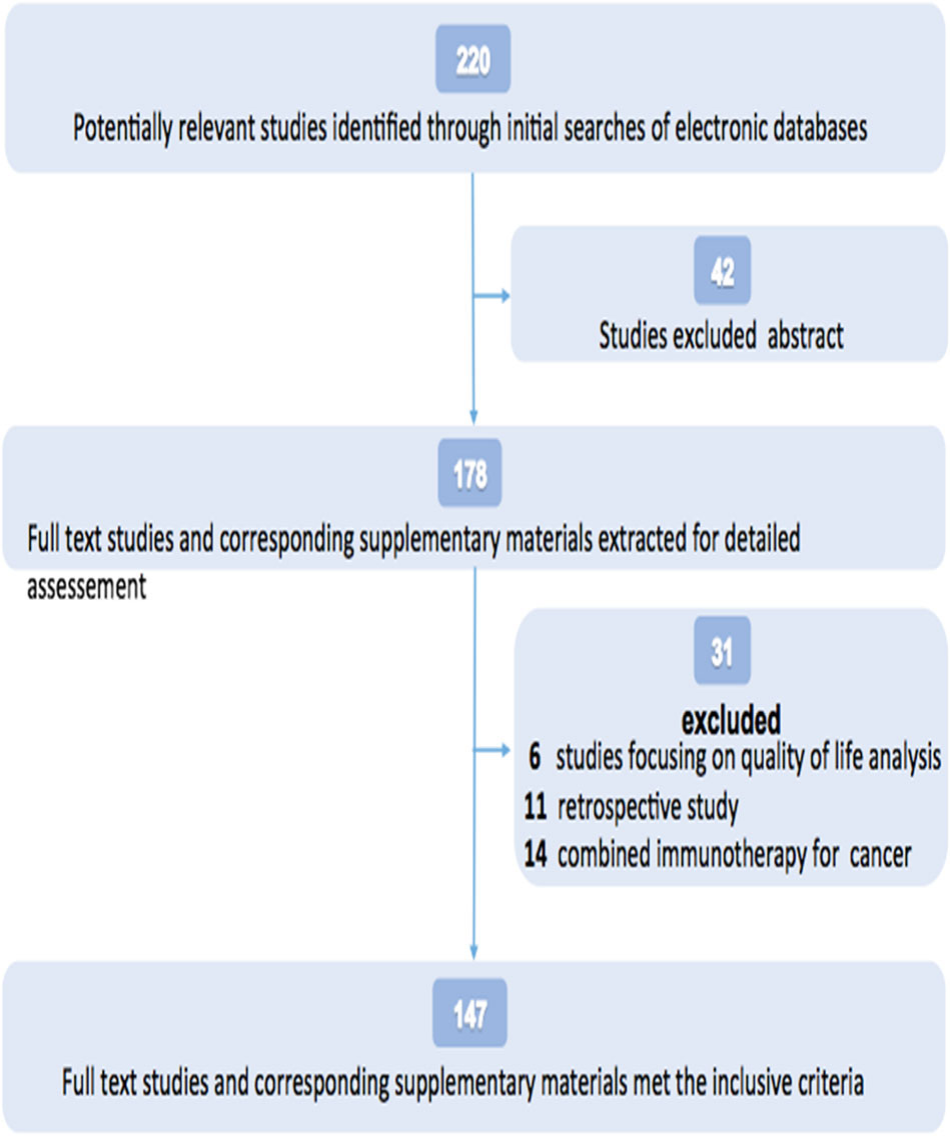
Flowchart of study selection and design.

The literature was first screened by reading the title and abstract and then some articles were removed if they were meeting abstracts, there was no inconsistent design, no access to the full text, and incomplete data. At the commencement of the study, a total of 220 related articles were identified, including 198 in PubMed, 12 in Embase and 10 in the web of Science, excluding 42 abstract papers, six quality of life analysis articles, 11 retrospective articles, 14 articles on immunocombination, and finally 147 cases of immunotherapy with a single drug to provide specific AE data of 23 761 patients (Fig 1).

### Basic characteristics of the literature

This analysis compared 11 different ICI‐related (grade 1–5 and 3–5) AEs, including a total of 147 studies and 23 761 patients.^5^, ^6^, ^7^, ^8^, ^9^, ^10^, ^11^, ^12^, ^13^, ^14^, ^15^, ^16^, ^17^, ^18^, ^19^, ^20^, ^21^, ^22^, ^23^, ^24^, ^25^, ^26^, ^27^, ^28^, ^29^, ^30^, ^31^, ^32^, ^33^, ^34^, ^35^, ^36^, ^37^, ^38^, ^39^, ^40^, ^41^, ^42^, ^43^, ^44^, ^45^, ^46^, ^47^, ^48^, ^49^, ^50^, ^51^, ^52^, ^53^, ^54^, ^55^, ^56^, ^57^, ^58^, ^59^, ^60^, ^61^, ^62^, ^63^, ^64^, ^65^, ^66^, ^67^, ^68^, ^69^, ^70^, ^71^, ^72^, ^73^, ^74^, ^75^, ^76^, ^77^, ^78^, ^79^, ^80^, ^81^, ^82^, ^83^, ^84^, ^85^, ^86^, ^87^, ^88^, ^89^, ^90^, ^91^, ^92^, ^93^, ^94^, ^95^, ^96^, ^97^, ^98^, ^99^, ^100^, ^101^, ^102^, ^103^, ^104^, ^105^, ^106^, ^107^, ^108^, ^109^, ^110^, ^111^, ^112^, ^113^, ^114^, ^115^, ^116^, ^117^, ^118^, ^119^, ^120^, ^121^, ^122^, ^123^, ^124^, ^125^, ^126^, ^127^, ^128^, ^129^, ^130^, ^131^, ^132^, ^133^, ^134^, ^135^, ^136^, ^137^, ^138^, ^139^, ^140^, ^141^, ^142^, ^143^, ^144^, ^145^, ^146^, ^147^, ^148^, ^149^ The interventions were: 46 pembrolizumab articles (*n* = 6598),^5^, ^6^, ^7^, ^8^, ^9^, ^10^, ^11^, ^12^, ^13^, ^14^, ^15^, ^16^, ^17^, ^18^, ^19^, ^20^, ^21^, ^22^, ^23^, ^24^, ^25^, ^26^, ^27^, ^28^, ^29^, ^30^, ^31^, ^32^, ^33^, ^34^, ^35^, ^36^, ^37^, ^38^, ^39^, ^40^, ^41^, ^42^, ^43^, ^44^, ^45^, ^46^, ^47^, ^48^, ^49^ 27 nivolumab articles (*n* = 3576),^49^, ^50^, ^51^, ^52^, ^53^, ^54^, ^55^, ^56^, ^57^, ^58^, ^59^, ^60^, ^61^, ^62^, ^63^, ^64^, ^65^, ^66^, ^67^, ^68^, ^69^, ^70^, ^71^, ^72^, ^73^, ^74^, ^75^ 13 atezolizumab articles (*n* = 2787),^76^, ^77^, ^78^, ^79^, ^80^, ^81^, ^82^, ^83^, ^84^, ^85^, ^86^, ^87^, ^88^ 12 avelumab articles (*n* = 3213),^89^, ^90^, ^91^, ^92^, ^93^, ^94^, ^95^, ^96^, ^97^, ^98^, ^99^, ^100^, ^101^, ^102^ 10 durvalumab articles (*n* = 1780),^103^, ^104^, ^105^, ^106^, ^107^, ^108^, ^109^, ^110^, ^111^ 22 ipilimumab articles (*n* = 4067)^5^, ^112^, ^113^, ^114^, ^115^, ^116^, ^117^, ^118^, ^119^, ^120^, ^121^, ^122^, ^123^, ^124^, ^125^, ^126^, ^127^, ^128^, ^129^, ^130^, ^131^, ^132^; eight tremelimumab articles (*n* = 1158),^133^, ^134^, ^135^, ^136^, ^137^, ^138^, ^139^, ^140^ three JS001 articles (*n* = 223),^141^, ^142^, ^143^ four SHR‐1210 articles (*n* = 178),^144^, ^145^, ^146^, ^147^ one sintilimab articles (*n* = 96)^148^ and one cemiplimab article (*n* = 85).^149^

### Meta‐analysis results

#### Meta‐analysis based on grade 1–5 adverse events of different ICIs in malignant tumors

**Figure 2:**
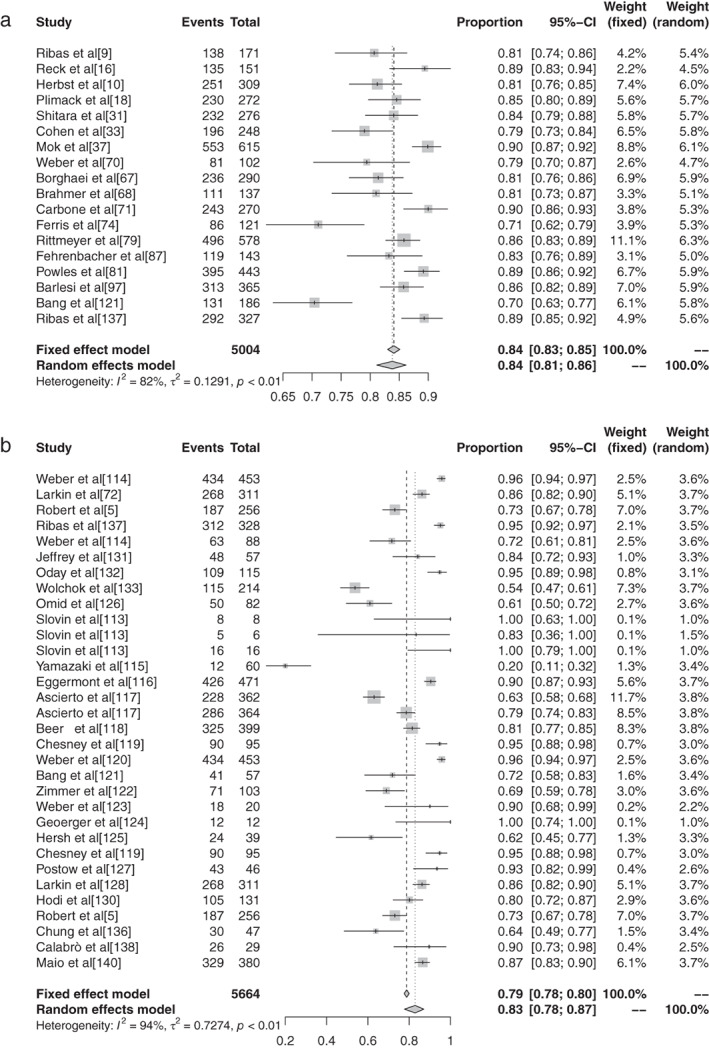
(**a**) Forest plot of conventional therapy; and (**b**) CTLA‐4 for grade 1–5 adverse events (AEs).

**Figure 3:**
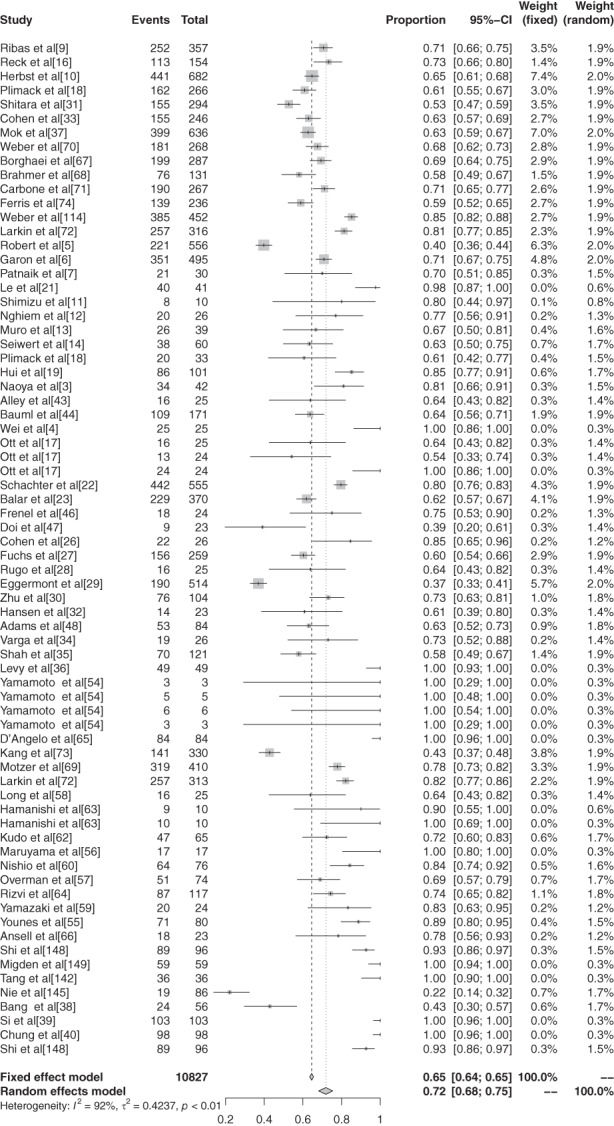
Forest plot of PD‐1 for grade 1–5 adverse events (AEs).

**Figure 4:**
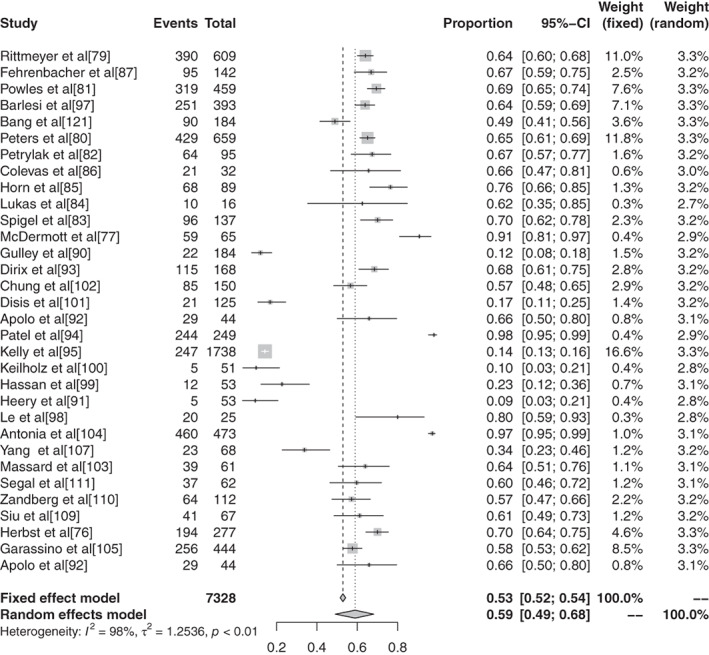
Forest plot of PD‐L1 for grade 1–5 adverse events (AEs).

**Figure 5:**
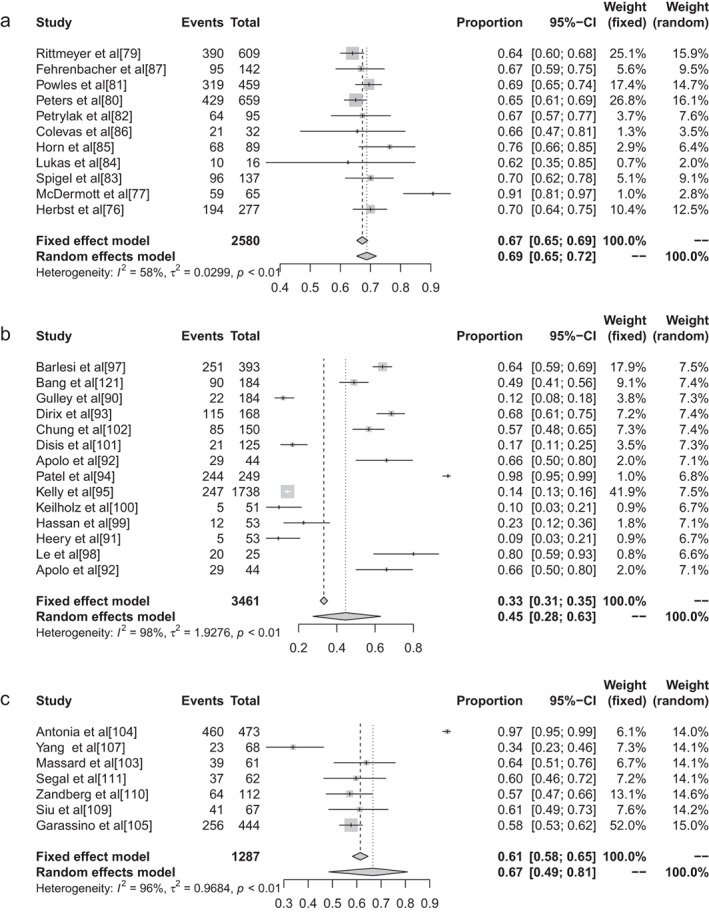
(**a**) Forest plot of atezolizumab; (**b**) avelumab; and (**c**) durvalumab or grade 1–5 adverse events (AEs).

The inverse variance method was used to analyze the incidence of grade 1–5 AEs of conventional therapy and three different immunological drugs CTLA‐4, PD‐1, and PD‐L1 to draw a forest plot. A total of 18 articles reported grade 1–5 AEs to conventional therapy. Heterogeneity analysis showed statistical heterogeneity among the results of each study (*P* < 0.1, I^2^ = 81.9%), so a random effects model was used. The results of meta‐analysis showed that the incidence of AEs was 83.81% (95% CI: 0.8113–0.8617, *P* < 0.1, I^2^ = 81.9%), and the forest plot is shown in Fig 2. A total of 32 articles reported CTLA −4 grade 1–5 AEs, heterogeneity analysis showed statistical heterogeneity between the results of each study (*P* < 0.1, I^2^ = 94.1%), so the random‐effects model was used for consolidation. Meta‐analysis showed that the incidence of AEs was 82.87% (95% CI: 0.7771–0.8704, *P* < 0.1, I^2^ = 94.1%), and the forest plot is shown in Fig 3. There were 72 reports which reported PD‐1 grade 1–5 AEs, and heterogeneity analysis showed that the statistical heterogeneity between the results of each study was greater (*P* < 0.1, I^2^ = 92.4%), so the random‐effects model was used for the combination. Meta analysis results showed that the reaction rate was 71.89% (95% CI: 0.6811–0.7539, *P* < 0.1, I^2^ = 92.4%), and its forest plot is shown in Fig 4. A total of 32 articles reported grade 1–5 AEs of PD‐L1 heterogeneity analysis indicating that the statistical heterogeneity between the results was greater (*P* < 0.1, I^2^ = 97.9%), so the random‐effects model was used for the combination. The meta‐analysis showed that the incidence of AEs was 58.95%. (95% CI: 0.4906–0.6817, *P* < 0.1, I^2^ = 97.9%), and the forest plot is shown in Fig 5.

#### Subgroup analysis of grade 1–5 adverse events based on different ICIs in malignant tumors

**Figure 6:**
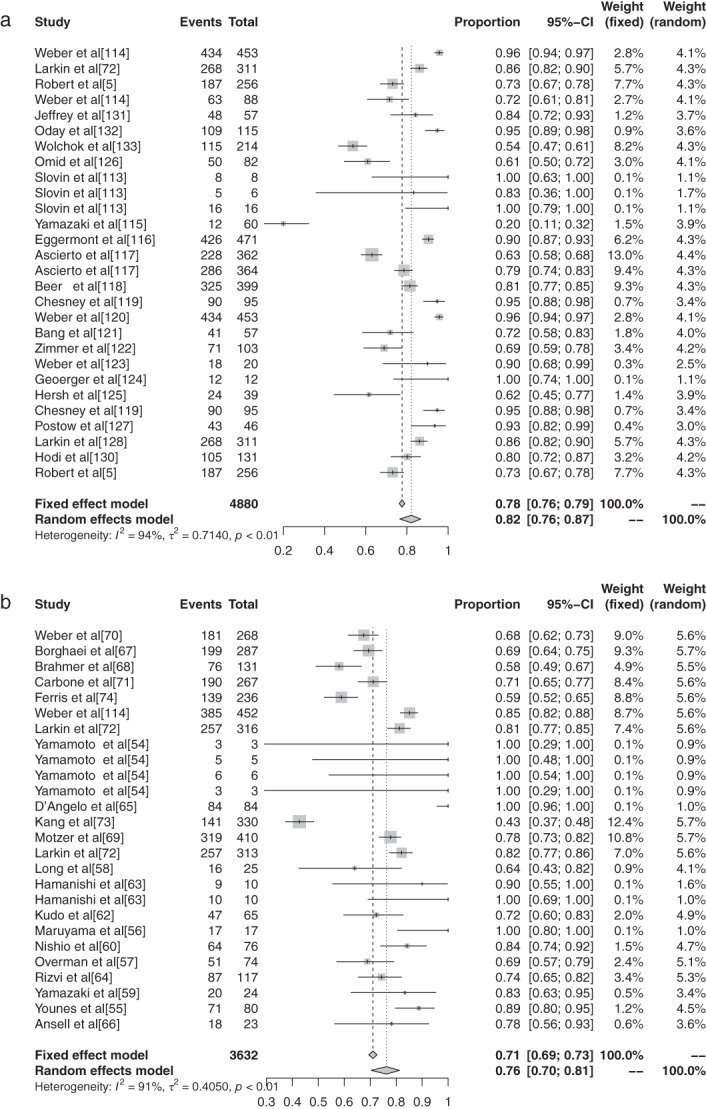
(**a**) Forest plot of ipilimumab; and (**b**) nivolumab for grade 1–5 adverse events (AEs).

**Figure 7:**
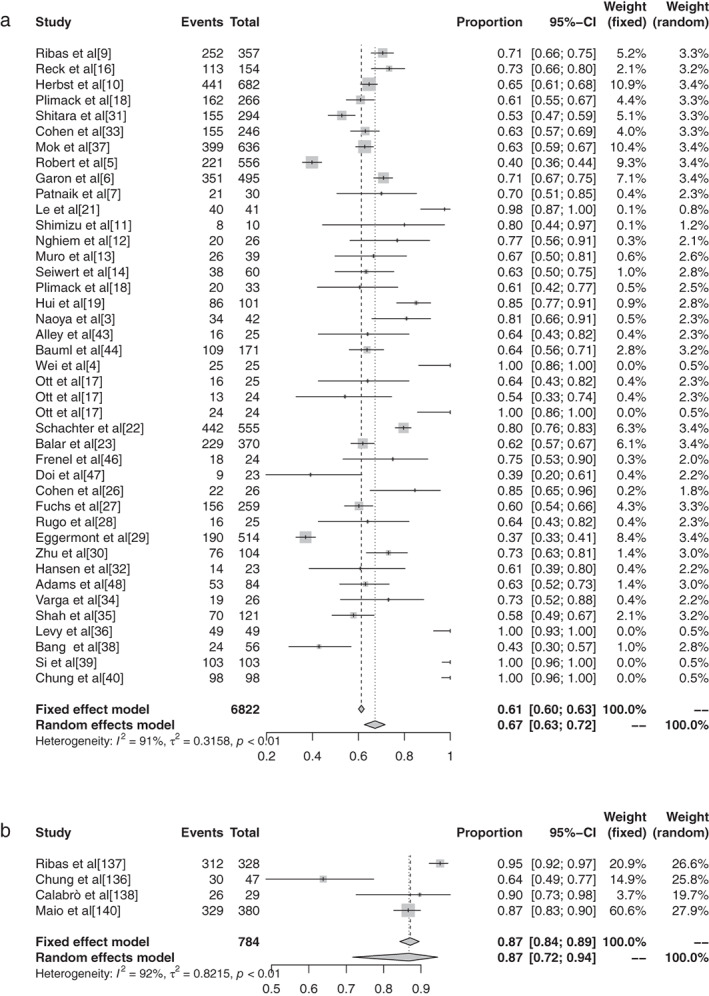
(**a**) Forest plot of pembrolizumab; and (**b**) tremelimumab for grade 1–5 adverse events (AEs).

**Figure 8:**
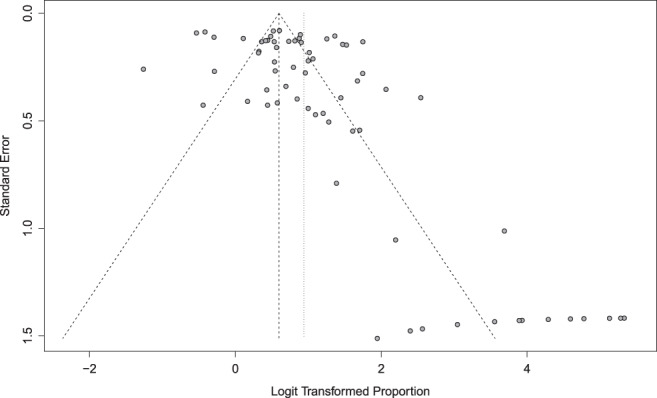
Funnel plot of PD‐1 for grade 1–5 adverse events (AEs).

**Figure 9:**
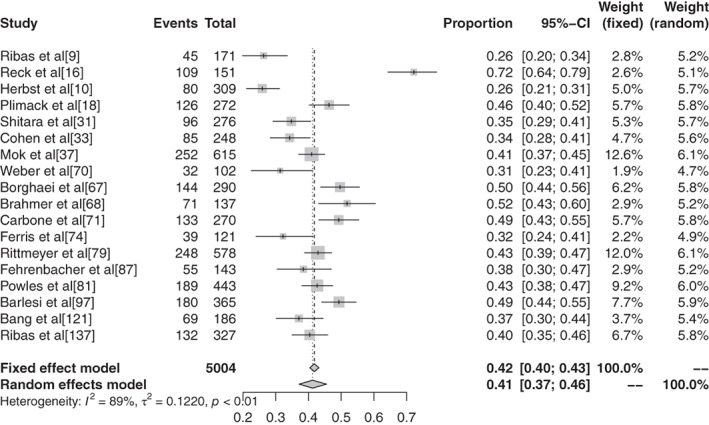
Forest plot of conventional therapy for grade 3–5 adverse events (AEs).

**Figure 10:**
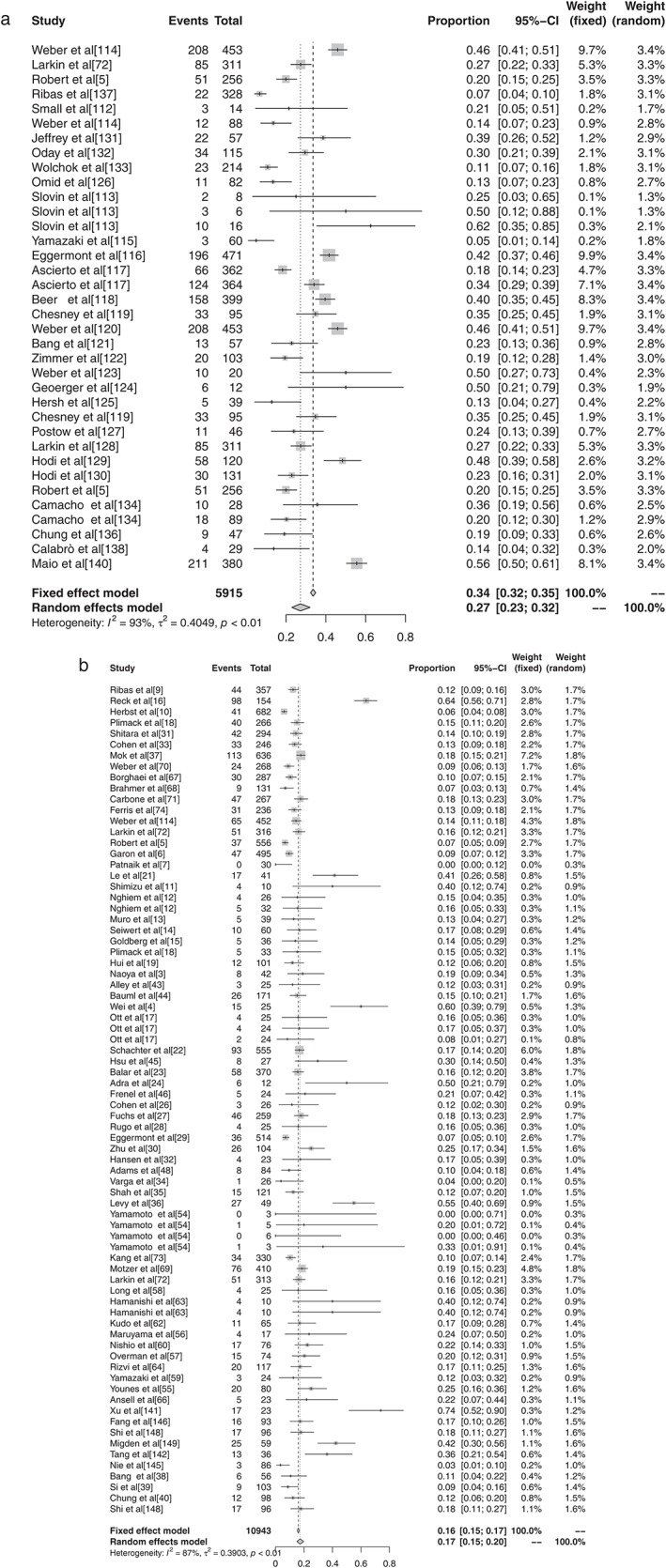
(**a**) Forest plot of CTLA‐4; and (**b**) PD‐1 for grade 3–5 adverse events (AEs).

**Figure 11:**
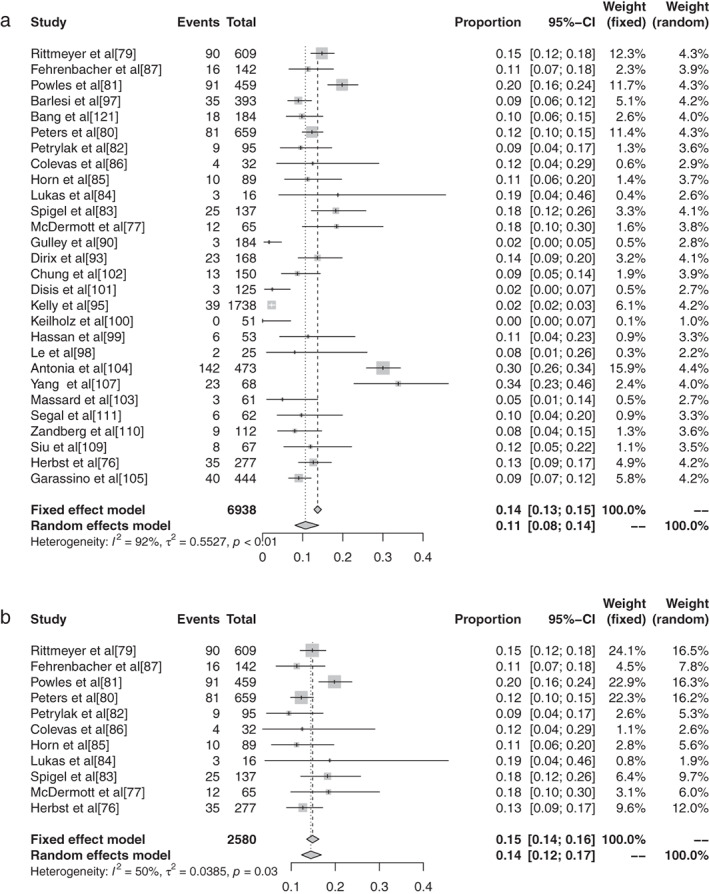
(**a**) Forest plot of PD‐L1; and (**b**) atezolizumab for grade 3–5 adverse events (AEs).

**Figure 12:**
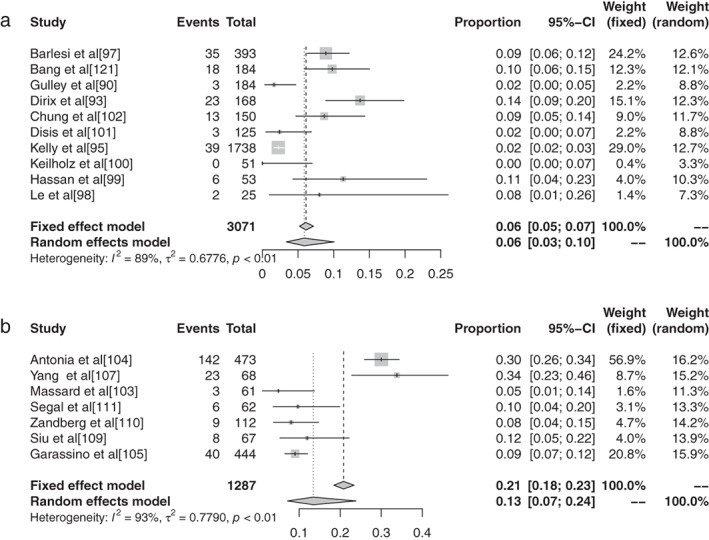
(**a**) Forest plot of avelumab; and (**b**) durvalumab for grade 3–5 adverse events (AEs).

There were 11 reports in the literature of grade 1–5 AEs to atezolizumab, and heterogeneity analysis showed statistical heterogeneity between studies was greater (*P* < 0.1, I^2^ = 57.7%), so the random effects model was used for the combination. The meta‐analysis showed that the incidence of AEs was 68.77% (95% CI: 0.6545–0.7190, *P* < 0.1, I^2^ = 57.7%), and the forest plot is shown in Fig 6. There were 14 reports of avelumab grade 1–5 AEs, and heterogeneity analysis showed greater statistical heterogeneity between the results of each study (*P* < 0.1, I^2^ = 98.3%), so the random effects model was used for the combination. The meta‐analysis showed that the incidence of AEs was 44.53% (95% CI: 0.2759–0.6285, *P* < 0.1, I^2^ = 98.3%), and the forest plot is shown in Fig 7. A total of seven articles reported durumumab grade 1–5 AEs, and heterogeneity analysis showed statistical heterogeneity among the results of each study was greater (*P* < 0.1, I^2^ = 95.9%), so the random effects model was used for the merger. Meta‐analysis results showed that the incidence rate of AEs was 66.63% (95% CI: 0.4855–0.8086, *P* < 0.1, I^2^ = 95.9%), and the forest plot is shown in Fig 8. There were 28 articles which reported ipilimumab grade 1–5 AEs, and heterogeneity analysis showed statistical heterogeneity among the results of each study (*P* < 0.1, I^2^ = 94.1%), so the random effects model was used for the merger. Meta‐analysis results showed the incidence of AEs was 82.18% (95% CI: 0.7649–0.8673, *P* < 0.1, I^2^ = 94.1%), and the forest plot is shown in Fig 9. A total of 26 articles indicated grade 1–5 AEs to nivolumab, and heterogeneity analysis showed greater statistical heterogeneity between the results of each study (*P* < 0.1, I^2^ = 90.7%), so the random effects model was used for the combination. Meta‐analysis showed that the incidence of AEs was 76.25% (95% CI: 0.7035–0.8129, *P* < 0.1, I^2^ = 90.7%), and its forest plot is shown in Fig 10. There were 41 articles which reported pembrolizumab grade 1–5 AEs, and heterogeneity analysis showed statistical heterogeneity among the results of each study (*P* < 0.1, I^2^ = 91.4%), so the random effects model was used for the combination. Meta‐analysis showed that its AE occurrence rate was 67.25% (95% CI: 0.6257–0.7161, *P* < 0.1, I^2^ = 91.4%), and its forest plot is shown in Fig 11. Four articles reported grade 1–5 AEs from tremelimumab, and heterogeneity analysis showed that the statistical heterogeneity between the study results was high (*P* < 0.1, I^2^ = 91.9%), so the random effects model was used for the combination. The meta‐analysis showed that the incidence of AEs was 86.78% (95% CI: 0.7172–0.9445, *P* < 0.1, I^2^ = 91.9%), and the forest plot is shown in Fig 12.

**Risk assessment of publication bias based on grade 1–5 adverse events in different ICIs in malignant tumors**

**Figure 13:**
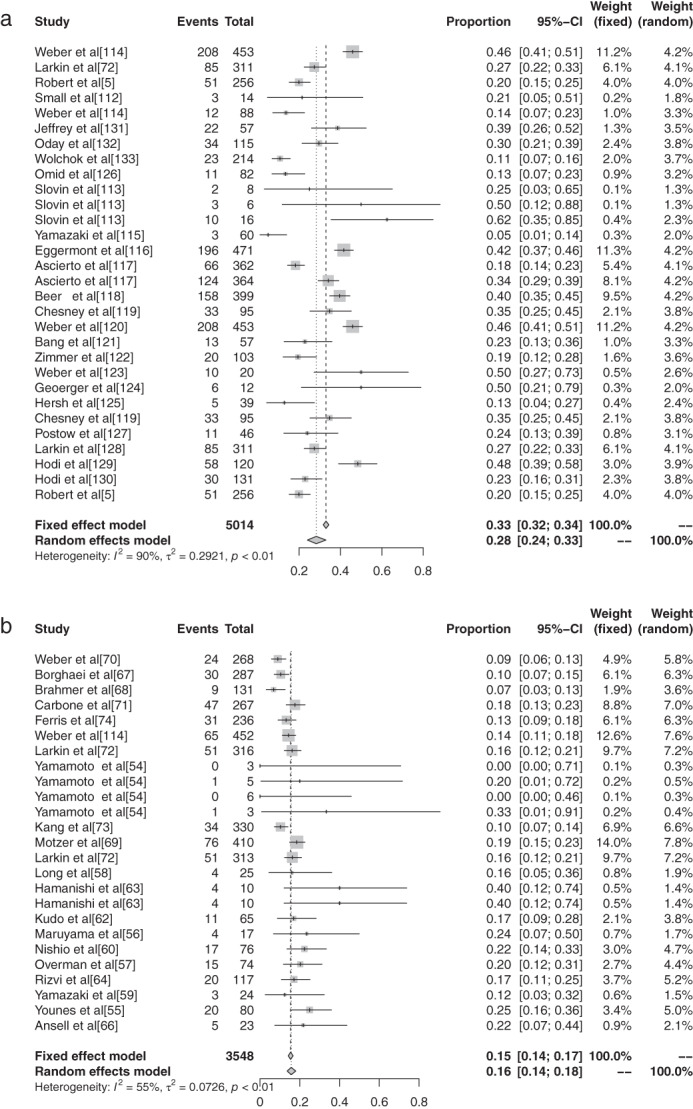
(**a**) Forest plot of ipilimumab; and (**b**) nivolumab for grade 3–5 adverse events (AEs).

All studies included in our analyses reported grade 1–5 AEs. To obtain a total overview, we performed a funnel plot. The points on the funnel plot were approximately symmetric, indicating that there was no publication bias. The *P* publication bias of the 18 studies included in conventional therapy was >0.05, and therefore no publication bias was found. The *P* publication bias of 32 studies included in CTLA‐4 was >0.05, and therefore no publication bias was found. The *P* publication bias of 32 studies included in PD‐L1 was >0.05, and therefore no publication bias was found. The *P* publications of 72 samples included in PD‐1 had a bias of *P* < 0.05, indicating that there was a risk of bias. The funnel plot is shown in Fig 13. In total, 11 articles reported grade 1–5 AEs to atezolizumab, and *P* publication bias was > 0.05, indicating that no publication bias was found.

A total of 14 articles reported that the avelumab grade 1–5 AEs *P* published bias was >0.05, indicating that no publication bias was found. There were seven articles which reported that the grade 1–5 AEs to durvalumab were unclear. In 28 articles, the grade 1–5 AEs to ipilimumab was reported to have had a *P* bias >0.05, indicating no publication bias was found. In 26 papers it was reported that grade 1–5 AEs to nivolumab *P* publication bias was >0.05, and therefore no publication bias was found. A total of 41 articles reported that the pembrolizumab grade 1–5 AEs *P* publication bias was >0.05, and no published bias was found. Four articles reported that the bias of grade 1–5 AEs to tremelimumab was unclear.

#### Meta‐analysis based on grade 3–5 adverse events of different ICIs in malignant tumors

**Figure 14:**
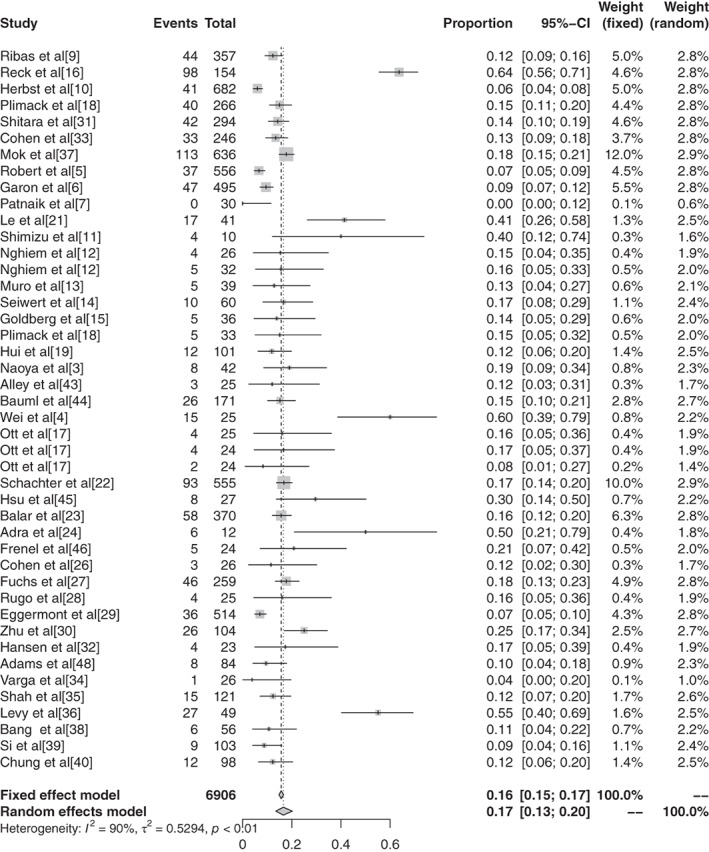
Forest plot of pembrolizumab for grade 3–5 adverse events (AEs).

**Figure 15:**
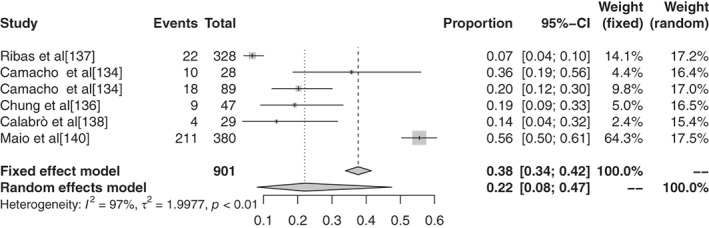
Forest plot of tremelimumab for grade 3–5 adverse events (AEs).

The inverse‐variance weighting method was used to analyze the incidence of grade 3–5 AEs of conventional therapy and three different immunological drugs ‐ CTLA‐4, PD‐1, and PD‐L1 ‐ and a forest plot was drawn. There were 18 grade 3–5 AEs to conventional therapy. Heterogeneity analysis showed statistical heterogeneity among the results of each study (*P* < 0.1, I^2^ = 88.7%), so the random effects model was used. The results of meta‐analysis showed that the incidence of AEs was 41.28% (95% CI: 0.3714–0.4555, *P* < 0.1, I2 = 88.7%), and its forest plot is shown in Figure 14. A total of 36 reports revealed CTLA‐4 grade 3–5 AEs, and heterogeneity analysis showed greater statistical heterogeneity between the results of each study (*P* < 0.1, I2 = 92.6%), so the random effects model was used for consolidation. Meta‐analysis showed that the incidence of AEs was 27.22% (95% CI: 0.2287–0.3204, *P* < 0.1, I2 = 92.6%), and its forest plot is shown in Fig 15. A total of 76 articles reported PD‐1 grade 3–5 AEs, and heterogeneity analysis showed that the statistical heterogeneity between the results of each study was greater (*P* < 0.01, I2 = 86.7%), so the random effects model was used for the combination. The meta‐analysis results showed that the incidence of AEs was 17.29% (95% CI: 0.1504–0.1979, *P* < 0.1, I^2^ = 86.7%). There were 28 articles which reported PD‐L1 grade 3–5 AEs, and heterogeneity analysis showed that the statistical heterogeneity between the results of each study was greater (*P* < 0.1, I^2^ = 92.1%), so the random effects model was used for the combination. The meta‐analysis showed that the incidence of AEs was 17.29% (95% CI: 0.0808–0.1397, *P* < 0.1, I^2^ = 92.1%).

#### Subgroup analysis of grade 3–5 adverse events based on different ICIs in malignant tumors

There were 11 articles which reported grade 3–5 AEs to atezolizumab. Heterogeneity analysis showed greater statistical heterogeneity among the results of each study (*P* < 0.1, I^2^ = 50.2%), so the random effects model was used for merging. The meta‐analysis showed that the incidence of AEs was 14.45% (95% CI: 0.1236–0.1684, *P* < 0.1, I^2^ = 50.2%). There were 10 articles which reported avelumab grade 3–5 AEs, and heterogeneity analysis showed greater statistical heterogeneity between the results of each study (*P* < 0.1, I^2^ = 88.9%), so the random effects model was used for consolidation. Meta‐analysis showed that the incidence of AEs was 5.86% (95% CI: 0.0335–0.1007, *P* < 0.1, I^2^ = 88.9%). Seven articles reported the grade 3–5 AEs to durvalumab, and heterogeneity analysis showed greater statistical heterogeneity between the results of each study (*P* < 0.1, I^2^ = 93.4%), so the random effects model was used for merging. Meta‐analysis results showed the incidence of AEs in durvalumab was reported in six articles to be 13.43% (95% CI: 0.0715–0.2383, *P* < 0.1, I^2^ = 93.4%). A total of 30 articles reported ipilimumab grade 3–5 AEs, and heterogeneity analysis showed larger statistical heterogeneity between the results of each study (*P* < 0.1, I^2^ = 90.4%), so the random effects model was used for the merger. Meta‐analysis results showed the incidence rate of its AEs was 28.27% (95% CI: 0.2401–0.3297, *P* < 0.1, I^2^ = 90.4%). A total of 25 articles reported grade 3–5 AEs to nivolumab. Heterogeneity analysis showed greater statistical heterogeneity among the results of each study (*P* < 0.1, I^2^ = 55.1%), so the random effects model was used for the merger. Meta‐analysis showed that the incidence of AEs was 15.72% (95% CI: 0.1368–0.1800, *P* < 0.1, I^2^ = 55.1%). A total of 44 articles reported grade 3–5 AEs to pembrolizumab, and heterogeneity analysis showed a greater statistical heterogeneity between the results of each study (*P* < 0.1, I^2^ = 52.94%), so the random effects model was used for consolidation. Meta‐analysis showed that the incidence of AEs was 16.58% (95% CI: 0.1347–0.2025, *P* < 0.1, I^2^ = 52.94%). Six articles reported grade 3–5 AEs to tremelimumab, and heterogeneity analysis showed a greater statistical heterogeneity between the results of each study (*P* < 0.1, I^2^ = 97.0%), so the random effects model was used for the merger. Meta‐analysis results showed that the AE rate was 22.04% (95% CI: 0.0812–0.4749, *P* < 0.1, I^2^ = 97.0%).

**Risk assessment of publication bias based on grade 3–5 adverse events in different ICIs in malignant tumors**

All studies included in this meta‐analysis reported grade 3–5 AEs. To fully reflect the situation, we performed a funnel plot analysis of the included papers. The points on the funnel plot were approximately symmetric, indicating that there was no publication bias. The *P* publication bias of the 18 articles included in the conventional therapy study was >0.05, and no publication bias was found. All 36 studies included in CTLA‐4 reported AEs, *P* publication bias was >0.05, and therefore no publication bias was found. The *P* publication bias of the 28 articles included in the PD‐L1 study was >0.05, and no publication bias was found. The *P* publication bias of the 76 articles included in the PD‐1 study was >0.05, and no publication bias was found. There were 11 articles which reported that atezolizumab had a bias of grade 3–5, AE published bias was >0.05, and no publication bias was found. There were 10 articles which reported grade 3–5 AEs to avelumab, *P* publication bias was >0.05, and therefore no publication bias was found. A total of 7 articles reported grade 3–5 AEs to durvalumab, and *P* publication bias was unclear. We found 30 reports in the literature of grade 3–5 AEs to ipilimumab, *P* publication bias was >0.05, and therefore no publication bias was found. There were 25 articles on nivolumab grade 3–5 AEs, publication bias was >0.05, and no publication bias was found. A total of 44 articles reported grade 3–5 AEs to pembrolizumab, *P* publication bias was >0.05, and no publication bias was found. There were six articles in which grade 3–5 AEs to temilimumab were reported, and the *P* publication bias was unclear.

### Discussion

This study compared 11 different ICI‐related (grade 1–5 and 3–5) AEs, including 147 articles and 23 761 patients (Table 1). There were 46 articles on pembrolizumab (*n* = 6598), 27 on nivolumab (*n* = 3576), 13 on atezolizumab (*n* = 2787), 12 on avelumab (*n* = 3213), 10 on durvalumab (*n* = 1780), 22 on ipilimumab (*n* = 4067), eight on tremelimumab (*n* = 1158), three on JS001 (*n* = 223), four on SHR‐1210 (*n* = 178), one on sintilimab (*n* = 96), and one on cemiplimab (*n* = 85). The grade 1–5 AEs were: 83.81% (conventional therapy), 82.87% (CTLA‐4), 71.89% (PD‐1), and 58.95% (PD‐L1). The rates in subgroup analysis were: 44.53% (avelumab), 66.63% (durvalumab), 67.25% (pembrolizumab), 68.77% (atezolizumab), 76.25% (nivolumab), 82.18% (ipilimumab), and 86.78% (tremelimumab). The grade 3–5 AE rates were: 41.28% (conventional therapy), 27.22% (CTLA‐4), 17.29% (PD‐1), and 17.29% (PD‐L1). The rates in subgroup analysis were: 5.86% (avelumab), 13.43% (durvalumab), 14.45% (atezolizumab), 15.72% (nivolumab), 16.58% (pembrolizumab), 22.04% (tremelimumab), and 28.27% (ipilimumab).

**Table 1:** Adverse reaction rate of different ICIs

Drug type	Pembrolizumab	Nivolumab	Atezolizumab	Avelumab	Durvalumab	Ipilimumab	Tremelimumab
No. of patients	6598	3576	2787	3213	1780	4067	1158
No. of studies	46	27	13	12	10	22	8
Grade 1–5 AE	67.25%	76.25%	68.77%	44.53%	66.63%	82.18%	86.78%
Grade 35 AE	16.58%	15.72%	14.45%	5.86%	13.43%	28.27%	22.04%

AE, adverse event.

ICI therapy has only been approved for a short period of time in China, and the clinical data is still at the collection stage. Reports of relevant domestic AEs are rare and cannot be combined. There have been three studies for JS001 which included 223 patients^143–145^. In the first study, 23 patients with advanced neuroendocrine tumors were included. Grade 1–5 AEs were not reported, and grade 3 AEs occurred in two patients (9%). There were no patients with grade 4 AEs. In the second study, 48 patients with nasopharyngeal carcinoma were enrolled. Of the 48 patients, 46 (96%) had mostly grade 1 or 2 AEs. A total of 17 cases (35%) had grade 3 or above AEs. However, it was not explicitly reported in the study whether all were AEs caused by treatment. In the third study, 36 patients with advanced melanoma or urinary tumors were enrolled, and 36 (100%) had grade 1–5 AEs, and the incidence of grade 3 and grade 3 AEs was 36%. SHR‐1210 included four studies and 178 patients^146–149^. The study involved classic Hodgkin's lymphoma, gastric/gastroesophageal junction adenocarcinoma, nasopharyngeal carcinoma, and solid tumor.

Of the 178 patients, 169 (94.9%) had grade 1–5 AEs, and 24 (13.5%) had grade 3–5 AEs. Sintilimab was included in only one study^148^ and out of 96 patients with classic Hodgkin's lymphoma, 89 (93%) had grade 1–5 AEs and 17 (18%) had grade 3–5 AEs. There was only one study on cemiplimab included (grade 1 and 2),^149^ and out of a total of 85 patients with cutaneous squamous cell carcinoma, 59 (69.4%) had grade 1–5 AEs, and 12 (14.1%) had grade 3–5 AEs.

The irAEs from ICI therapy are mainly caused by immune activation against the autoimmune system. Other secondary toxicity may be due to changes in immune cell function and cytokines released by immune and nonimmune cells (including tumor cells and their microenvironment). irAEs can affect any organ system, and the implementation of early diagnosis and early intervention is critical to patient safety and is the key to the management of irAEs. Patients should be educated to report an irAE to their oncologist as soon as possible, even if the intensity of their symptoms is not serious. The typical treatment for irAEs is to suspend or continue ICI therapy, hospitalize if necessary, rule out the cause of infection, use steroids orally or intravenously, gradually reducing the dose over a period of several weeks. If hormone therapy is not effective, consideration should be given to using mycophenolate or infliximab (if there is no liver damage), and an affected organ specialist should be consulted if necessary. The patients included in the above statistics had no evidence of pre‐existing autoimmune diseases.

It becomes very challenging when oncologists must make decisions among many therapies with similar efficacy and/or specific toxicity characteristics. The advantage of meta‐analysis in this study is that it may reduce publication bias and identify obvious results with higher efficiency. Due to the combination of smaller and larger studies, the effective sample size will be greatly increased. The results of this meta‐analysis can help oncologists choose the type of ICI when deciding on an ICI plan and when planning to use ICIs for future research.

ICIs are effective against a variety of cancers. Compared with CTLA‐4, PD‐1 and PD‐L1 have a lower incidence of iRAEs and still have clinical therapeutic effects. The limitations are as follows: single‐agent different dose in immunotherapy treatment may have an impact on the outcome; most of the literature in this study is overseas; ICI‐related domestic research does not show obvious advantages; the quality of the included research is lower; and there are few relevant studies. In the future, multicenter, large sample, and high quality research will be needed to further support and verify the findings of this study.
